# Challenges to implement laparoscopic appendectomy as the first-line treatment for acute appendicitis: a fifteen-year experience in a tertiary hospital in Brazil

**DOI:** 10.1590/0100-6991e-20233527-en

**Published:** 2023-05-04

**Authors:** SERGIO HENRIQUE BASTOS DAMOUS, CARLOS AUGUSTO METIDIERI MENEGOZZO, MARCELO CRISTIANO ROCHA, FRANCISCO SALLES COLLET-E-SILVA, EDIVALDO MASSAZO UTIYAMA

**Affiliations:** 1 - Hospital das Clínicas da Faculdade de Medicina da Universidade de São Paulo, Cirurgia - São Paulo - SP - Brasil

**Keywords:** Laparoscopy, Laparoscopic Appendectomy, Appendicitis, Medical Education, Laparoscopia, Apendicectomia, Apendicite, Apêndice, Educação Médica

## Abstract

**Background:**

the barriers to implement emergency laparoscopy in public teaching hospitals involve issues such as resident learning curves and resource costs and availability. This study was designed to describe the issues facing the implementation of laparoscopic approach for acute appendicitis over 15 years in a single academic center in Brazil.

**Materials and Methods:**

retrospective study of patients undergoing emergency appendectomy from 2004 to 2018. Clinical data were compared to four major actions implemented in the emergency surgical service: minimally invasive surgery training for residents (2007), laparoscopic stump closure using metal clips (2008), 24/7 availability of laparoscopic instruments for emergency surgeries (2010), and third-party contract for maintenance of the laparoscopic instruments and implementation of polymeric clips for stump closure (2013). We evaluated the increase in laparoscopic appendectomy after the implementation of those major changes.

**Results:**

we identified 1168 appendectomies during the study period, of which 691 (59%), 465 (40%), and 12 (1%) were open, laparoscopic, and converted, respectively. The implementation of the major changes since 2004 resulted in an increase of laparoscopic appendectomies from 11% in 2007 to 80% in 2016. These actions were decisive in the widespread use of laparoscopy for acute appendicitis (p<0.001). The standardization of the hem-o-lok clip in the treatment of the appendiceal stump made the procedure more feasible, reducing the surgical time using laparoscopic access and increasing the team’s adherence, so that this became the route of choice in about 85% of cases in the period from 2014 to 2018, 80% performed by 3^rd^ year resident physicians. No intraoperative complications were noted related to laparoscopic access, even in more complicated appendicitis. There was no mortality reported, no reoperations or readmissions to hospital during a 30-day postoperative period.

**Conclusion:**

the development of a feasible, reproducible, and safe technical standardization, associated with continuous cost optimization, are the cornerstones for a consistent and viable change in the current practice for appendectomies in middle and lower-income countries.

## RESUMO

**Introdução:**

o presente estudo tem como objetivo avaliar as medidas que permitiram a implementação de cirurgias laparoscópicas de urgência em hospital de ensino médico ao longo de 15 anos.

**Método:**

foi realizado estudo clínico retrospectivo de pacientes submetidos a Apendicectomia aberta ou Laparoscópica no período de 15 anos (2004 a 2018). Os dados clínicos foram confrontados com as ações implementadas: 1) Treinamento dos médicos residentes em cirurgia minimamente invasiva (2007); 2) Uso do *endoclip* para tratamento do coto apendicular (2008); 3) Disponibilidade do set de laparoscopia em tempo integral para o Pronto Socorro (2010); e, 4) Terceirização do serviço de manutenção do set e materiais de vídeo e uso do endoclip Hem-o-lok^®^ para tratamento do coto apendicular (2013).

**Resultados:**

foram realizadas 1.168 cirurgias, das quais 691 abertas (59%), 465 laparoscopias (40%) e 12 convertidas (1%). O treinamento da equipe levou à realização de 11% de laparoscopia (2007) e o uso do endoclip para tratamento do coto apendicular para 16% (2008). Com a disponibilização do set de vídeo em tempo integral para o Pronto Socorro a partir de 2011, houve aumento de laparoscopias para 26% (2012). A padronização do endoclip Hem-o-lok^®^ no tratamento do coto apendicular aumentou para 85% e a partir de 2016 foi realizado em todos os casos, sem complicação relacionada ao seu uso, mesmo em apendicites complicadas.

**Conclusão:**

o emprego da laparoscopia no serviço de urgência envolve além do treinamento da equipe cirúrgica, investimento tecnológico contínuo e uso de técnicas que sejam factíveis, viáveis e seguras.

## INTRODUCTION

Use of laparoscopic appendectomy has increased in the last decade in relation to the conventional (open) technique, as it is considered viable and safe for both simple and complicated cases. It is indicated by several studies as the preferred access route due to lower morbidity and mortality and hospitalization time and better postoperative recovery, without an increase in the incidence of abscess, compared to the open route^1^ ^-^ ^4^. 

Although it is considered by some to be the gold standard for the treatment of obese and reproductive-age women^5^ ^,^ ^6^, some challenges still need to be overcome for there to be consensus on its use in the general population, such as the cost and learning curve of the surgical team. A prospective multicenter study involving 44 countries over a 6-month period showed similar rates of laparoscopic and open appendectomy - 51.7% and 42.2% respectively^7^. Laparoscopic access is common in high income countries (87.7%) but infrequent in lower income countries (8.1%)^8^. Although Brazil is an upper middle-income economy, it is one of the most unequal countries in the world, as a significant amount of people liver under poverty. In 2019, only 6.3% of appendectomies were laparoscopic, and the state of São Paulo, which is considered one of the most developed in the country, socially and economically, was responsible for 10% of the laparoscopic appendectomies^9^. These data exemplify how heterogenous the different regions of Brazil are. 

The challenges of implementing laparoscopy in urgent care in public services in developing countries such as Brazil range from the learning curve of resident physicians to the cost and availability of video equipment and consumables. When a healthcare system is struggling to deliver basic surgical procedures, the introduction of a more complex intervention must be considered carefully, even with the broad advantages of laparoscopic approach^8^. 

The use of the clip in the treatment of the appendicular stump was a great contribution to the feasibility of the laparoscopic technique, as it added technical ease and reduced complication rates and cost optimization compared to the use of staplers^10^ ^,^ ^11^. 

In the past decade, laparoscopic appendectomy performed by resident physicians under supervision was considered a safe technique, with a reduction in complications as the learning curve increased^12^. In this context of medical education in minimally invasive surgery, the present study aims to evaluate the impact of actions implemented in the emergency department of a public hospital in Brazil, which allowed the implementation of the use of laparoscopy as a routine in the treatment of acute appendicitis, describing its increasing usage over 15 years. The secondary outcomes are to compare the results of laparoscopic and open appendectomy in terms of patient epidemiology, surgery time, postoperative complications, and length of hospital stay.

## PATIENTS AND METHODS

A retrospective clinical study was carried out by analyzing the medical records of patients who underwent open or laparoscopic appendectomy at the Division of Clinical Surgery III (DCC III) of the Hospital das Clínicas da Faculdade de Medicina da Universidade de São Paulo (HC/FMUSP) from January 2004 to December 2018, after approval by the Ethics Committee for the Analysis of Research Projects (CAPPesq), Number 5.234.925. 

The Hospital das Clínicas da Faculdade de Medicina da Universidade de São Paulo is the largest hospital complex in Latin America and a reference for highly complex cases of different surgical specialties. The emergency sector has 5 assistant physicians and 12 General Surgery resident physicians every 24 hours. Resident physicians are distributed in the sector according to the year of the internship, as follows: 1 from the 1^st^ year, 3 from the 2^nd^ year, 1 from the 3^rd^ year and 1 from the 4^th^ year. Appendicitis cases arrive at the service transferred from basic health units via the state’s bed regulation system. 

In 2007, the laparoscopy training center in animal models (pigs) was opened for resident physicians with 4 arenas and 2 virtual simulators, becoming part of the mandatory internship for resident physicians in the emergency department, with a workload of 3 hours/week. This training center was also offered to attending physicians who were not trained in laparoscopic access. 

The following data were collected: patient’s age, presence of comorbidities (ASA class - American Society of Anesthesiology), duration of surgery, qualification of the main surgeon (resident or assistant physician), treatment used in the appendicular stump (ligature/suture with surgical threads, clips or endostapler), appendix histological grading, postoperative day of discharge and postoperative complications. Clips include metallic or polymer (hem-o-lok) devices. The postoperative complications were evaluated during patient follow-up at the outpatient surgical department one month after the surgery.

Such clinical parameters were confronted on the timeline with the following actions implemented:

1. Minimally invasive surgery training for residents (2007);
2. Laparoscopic stump closure using metal clips (2008);
3. Full-time availability of laparoscopic instruments for emergency surgeries (2011); and
4. Outsourcing for maintenance of the laparoscopic instrument and implementation of polymeric clips for stump closure (2013).

### Statistical analyses

All statistical analyses were performed using Graphpad Prism 9.1 (Graphpad Software Inc., CA, USA). P values lower than 0.05 were considered significant. The comparison of the operative time between the groups was performed using the Mann-Whitney test after analysis of normality using the Shapiro Wilk test. Results are expressed as median-interquartile range. Timeline comparisons were performed using the Chi-Square test for tendency. The analysis of temporal trends was performed using the simple linear regression method (y=β0+β1*x1), where y corresponds to the occurrence values; x to time; β0 to the intersection between the line and the vertical axis and β1 to the slope of the line; and a significance level of 5% and 95% CI was considered. When p<0.05, increasing trends were considered when β1 was positive and decreasing when β1 was negative.

## RESULTS

In the 15-year period (2004 to 2018), 1,168 appendectomies were performed, of which 691 were open (59%), 465 by laparoscopy (40%) and 12 were video-assisted (converted) (1%). The patients were more often males (57%) and median aged 38 years old. The median surgical time was 105 minutes (70-123) for the open access and 120 minutes (90-133) for the laparoscopic access (p<0.05, Table I). Data regarding operative time, hospital stay, reoperations, comorbidities (ASA class) and appendix histological grading are presented in Table I. The mean length of hospital stay, and reoperation rate were comparable in both groups (p>0.05). Most patients were preoperatively classified as ASA class I or II. The most frequent type of inflammation was phlegmonous ulcer appendicitis.

**Table 1:** Operative time, hospital stay, reoperation rate, comorbidities (ASA class) and appendix histological grading of patients undergoing conventional (open access) or laparoscopic appendectomy in the emergency.

	Open access	Laparoscopic access
Operative time (15-year period)*	105 (70-123)	120 (90-133)*
Hospital stay	2 days	1,5 days
Reoperation	2.1%	2%
ASA		
I	85%	83%
II	12%	13%
III	3%	4%
Appendix histological grading		
Mucocele	0.3%	0
Lymphoid hyperplasia	5.7%	2%
Phlegmonous	26%	27%
Phlegmonous ulcer	50%	55%
Gangrenous	18%	14%

*p≤0.0010 vs. open access, Mann-Whitney test; Operative time are expressed as median-interquartile range; Hospital stay: post-operative day of discharge; ASA: American Society of Anesthesiology.

**Figure 1:**
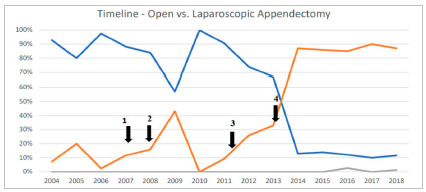
Analysis of the actions performed in an emergency department of a public hospital in Brazil during a 15-year period (2004 to 2018) for the implementation of laparoscopic access in appendectomies. 1 - Minimally invasive surgery training for residents (2007) → 16% laparoscopic appendectomies (2008); 2 - Laparoscopic stump closure using metal clips (2008) → 43% laparoscopic appendectomies (2009); 3 - Full-time availability of laparoscopic instruments for emergency surgeries (2011) → 26% laparoscopic appendectomies (2012); 4 - Third-party contract for maintenance of the laparoscopic instrument and implementation of polymeric clips for stump closure (2013) → 87% laparoscopic appendectomies (2014). Timeline comparisons were performed using the Chi-Square test for tendency, p<0.001.

Staff training allowed 11% of laparoscopy to be performed in 2007. In the following year (2008), a metallic clip began to be used as an option for the treatment of the appendicular stump, facilitating the technique and leading to an increase of 16% usage of laparoscopic access. In 2010, no laparoscopy was performed because there was no video set available for urgent surgeries, which also had an impact in 2011 (9.3% of surgeries used laparoscopy). As of 2011, a full-time video set was made available for emergency surgeries, with an increase in laparoscopic surgery to 26% (2012), but the unavailability of materials such as endostapler and equipment maintenance service were still limiting factors (Figure 1).

**Figure 2:**
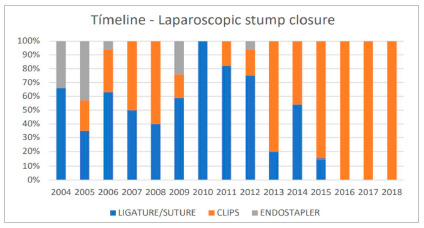
Laparoscopic stump closure in an emergency department of a public hospital in Brazil during a 15-year period (2004 to 2018). Clip: LT 400 until 2012, hem-o-lok clips from 2013 onwards. Chi-Square for trend test, p=0.0020.

The actions taken from 2013 onwards were decisive for the implementation of laparoscopic access in the service (p<0.001). The standardization of the hem-o-lok clip in the treatment of the appendiceal stump made the procedure more feasible even in appendices with wide bases, reducing the surgical time using laparoscopic access and increasing the team’s adherence, so that this became the route of choice in about 85% of cases in the period from 2014 to 2018 (Figure 1) and 80% of the time was performed by 3^rd^ year resident physicians. In the same year of standardization of the hem-o-lok clip (2013), it was used in 80% of cases vs. 20% of the ligation/suture and no cases with a stapler, and from 2016 onwards the treatment of the stump with hem-o-lok clips started to be performed in all cases (Figure 2). No intraoperative complications were noted related to its use, even in more complicated appendicitis. There was no mortality reported. There were no reoperations or readmissions to hospital during a 30-day postoperative period in our study patients.

**Figure 3:**
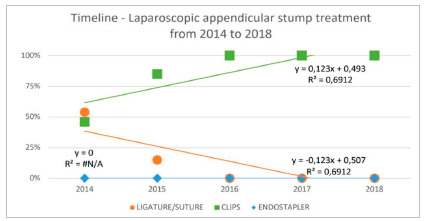
Laparoscopic stump closure in an emergency department of a public hospital in Brazil in the period from 2014 to 2018. Clip = hem-o-lok clips. Simple linear regression method.

**Figure 4:**
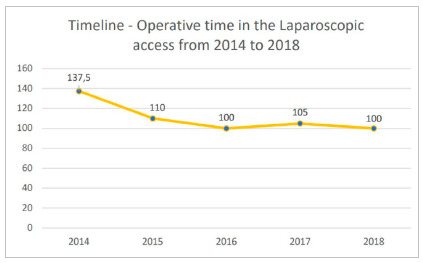
Laparoscopic appendectomy operative time (in minutes) in an emergency department of a public hospital in Brazil from 2014 to 2018. Data are expressed as median. Nonlinear regression method, R square=0.7293.

The analysis of temporal trends was evaluated from 2014 to 2018, in the three types of appendicular stump treatment. Figure 3 presents the temporal trends: positive for the use of clip, negative for the use of ligature or suture and an absence of activity using the endostapler. When analyzing the last years from the last action implemented (2014-2018), the median operative time for the laparoscopic access was shorter in this period, 105 minutes (89-125) and similar to the open access (p>0.05) but with a tendency to reduce the laparoscopy route in this five-year period (R^2^=0.7293, Figure 4).

## DISCUSSION

The present study shows the impact of the actions implemented to make laparoscopic access the preferred route for the treatment of acute appendicitis in the emergency department of a public hospital in Brazil. The challenges facing laparoscopy vary by countries and types of hospitals. The state of São Paulo is considered one of the most developed in the country, with a human development index equivalent to that of developed countries (0.783 in 2019) and the highest per capita income in the country (32% of the total)^13^. Even in this scenario, for the implementation of laparoscopic access in the emergency department of a public hospital, it was necessary to overcome barriers common to any developing country. In the public hospitals in Brazil, as well as in lower income countries, the greatest barrier to widespread adoption of laparoscopic access has been cost. In addition to training the assistance team, it is necessary to ensure engineering staff undertake equipment maintenance and establish affordable supply chains^14^ ^,^ ^15^.

Although laparoscopic appendectomy is considered a basic procedure, the main difficulty in performing it involves the treatment of the appendicular stump. The endostapler is considered the universal device for this purpose, as it can be used in appendicitis of different degrees of severity, but its high cost makes its routine use in public service unfeasible. Thus, to enable laparoscopic access for appendectomies in the service, the only option for the treatment of the appendiceal stump was ligature or suture with surgical thread, performed with difficulty with the use of three punctures, especially for physicians beginning their training. Although the type of stump closure is not the objective of this study, its standardization with faster techniques (clips vs suture) had a direct impact on the adherence of the medical team and, consequently, on the increase in laparoscopic appendectomies, the main outcome of this work.

In our service, the use of devices such as metal clips made laparoscopic access much simpler, as it allowed its application quickly and safely, with the use of only three punctures - one used for optics, another for appendicular traction and presentation of the base and the third to seal the stump with the clip. Therefore, the use of the metallic clip in 2008, associated with the training of the entire team, led to an increase of laparoscopic access for appendectomies to 43%. 

A limiting factor for the use of the metallic clip was the cases of appendix with a wide base or with necrosis (up to 1/3 of the cases of complicated appendicitis), in which there is a risk of clip opening and sliding off^16^. To overcome this limitation, in 2013, the hem-o-lok clip was standardized in the service, which proved to be safe and viable, both from a technical and financial point of view, in accordance with the literature^11^ ^,^ ^17^ ^-^ ^19^. In addition to the lower cost, clinical trials have shown that the surgical time of using the hem-o-lok clip is the same as that of the endostapler^17^.

The longer surgical time of the laparoscopic access compared to the open approach is another point to be highlighted, as it also impacts the occupation of rooms in the operating room and higher costs for anesthesia due to increased time but tends to decrease with experience^14^. Our median operative time for laparoscopic access was 120 minutes over 15 years. Previous studies show a significant reduction in operative time with mechanical devices compared with ligature-based techniques^11^. In our study, when considering the last 5 years (use of the hem-o-lok clip), there was a reduction to 105 minutes. This time is similar to that of older studies and longer than more recent studies^16^ ^-^ ^18^ which is a limitation of our study. However, in the more recent studies, the procedures were performed by trained surgeons, different from our study carried out in a teaching hospital, in which laparoscopic appendectomy became a procedure mostly performed by 3^rd^ year resident physicians as the main surgeon. 

The lack of full time availably of equipment and resources meant that no appendectomy was performed in 2010. Laparoscopy materials are generally prioritized for elective procedures, and their use in emergencies is restricted to periods when they were not used for electives and only during business hours, greatly limiting their use. To solve this problem, in 2011 our service acquired a video set for exclusive use in emergencies, with full-time availability. This action allowed the return of laparoscopic appendectomies as in previous years, but it was not enough for this access to become preferred, as the lack of specialized maintenance service for the equipment still impacted its use. 

Laparoscopic access requires continuous investment for equipment maintenance and acquisition of new technologies/devices. This challenge is even greater in the 24-hour public emergency service, with surgeries at different times, and the maintenance service must accompany this full-time operation, making it, in operational terms, more complex than other sectors with predictable hours and surgeries. A multicenter study with 52 countries followed the management of appendicitis in high-, middle-, and low-income countries over 6 months. They noted that there were more operations through the night in low and middle HDI countries in comparison to high HDI countries^8^. 

Accessing and maintaining equipment have been highlighted as barriers to sustainable implementation of laparoscopic training in in low- and middle-income countries. Potential solutions require collaboration between multiple stakeholders, which is often influenced by local sociocultural factors. This can only be addressed through guidance and policy from professional and governmental bodies, incorporating laparoscopic surgery in all surgery training curricula^14^ ^,^ ^15^. The outsourcing of the video set maintenance service and its respective materials was decisive in our service. This action, associated with the standardization of the appendicular stump treatment technique with a hem-o-lok clip, allowed the laparoscopic access to surpass the open route in the year following its implantation, and consolidating its routine use in subsequent years. For long-term planning for sustainability, maintenance facilities must be established to ensure cost-effectiveness. 

This work contributes to medical education in minimally invasive surgery, showing which barriers were overcome over 15 years for the implementation of laparoscopy in the emergency sector, even in lower income countries. After 10 years of investment, laparoscopy has become the preferred access route for appendectomies, maintaining this result sustained in the last 5 years of analysis, being a procedure performed mostly by resident physicians. The major strength of this study is to improve outcomes in surgery, allowing multiple generations of surgeons to incorporate laparoscopy into their daily practice. 

## CONCLUSION

The development of a feasible, reproducible, and safe technical standardization, associated with continuous cost optimization, are the cornerstones for a consistent and viable change in the current practice for appendectomies in middle and lower-income countries.
